# Topic Modeling Analysis of Children’s Food Safety Management Using BigKinds News Big Data: Comparing the Implementation Times of the Comprehensive Plan for Children’s Dietary Safety Management

**DOI:** 10.3390/foods14152650

**Published:** 2025-07-28

**Authors:** Hae Jin Park, Sang Goo Cho, Kyung Won Lee, Seung Jae Lee, Jieun Oh

**Affiliations:** 1 Department of Nutritional Science and Food Management, Ewha Womans University, Seoul 03760, Republic of Korea; jin96@ewhain.net; 2 Department of Big Data, Kyungbok University, Namyangju-si 12051, Republic of Korea; ancestor9@kbu.ac.kr; 3 Department of Nutritional Home Economics Education, Korea National University of Education, Cheongju-si 28173, Republic of Korea; kwlee@knue.ac.kr; 4 Department of Food Science and Nutrition, Yongin University, Yongin 17092, Republic of Korea; sjlee@yongin.ac.kr; 5 College of Science & Industry Convergence, Ewha Womans University, Seoul 03760, Republic of Korea

**Keywords:** children, food safety, Comprehensive Plans for Safety Management of Children’s Dietary Life, BigKinds, LDA

## Abstract

As digital technologies and food environments evolve, ensuring children’s food safety has become a pressing public health priority. This study examines how the policy discourse on children’s dietary safety in Korea has shifted over time by applying Latent Dirichlet Allocation (LDA) topic modeling to news articles from 2010 to 2024. Using a large-scale news database (BigKinds), the analysis identifies seven key themes that have emerged across five phases of the national Comprehensive Plans for Safety Management of Children’s Dietary Life. These include experiential education, data-driven policy approaches, safety-focused meal management, healthy dietary environments, nutritional support for children’s growth, customized safety education, and private-sector initiatives. A significant increase in digital keywords—such as “big data” and “artificial intelligence”—highlights a growing emphasis on data-oriented policy tools. By capturing the evolving language and priorities in food safety policy, this study provides new insights into the digital transformation of public health governance and offers practical implications for adaptive and technology-informed policy design.

## 1. Introduction

Children’s diet is an important determinant of healthy growth and development, and this has been highlighted as a social issue both domestically and internationally [1]. Recent technological advancements and environmental changes have had a significant impact on the dietary environment of children, creating new trends, such as the expansion of online food purchases, alterations in the consumption environment centered on convenience stores, and the introduction of personalized services using artificial intelligence (AI) and big data [2,3]. These changes have a direct impact on children’s eating habits, food choices, and nutritional status, and countries are responding with a variety of policies through public–private partnership.

For example, in the United States the Healthy Corner Stores Network (HCSN) initiative, launched in 2014, installed refrigerators and display units for nutritious foods in small stores, complemented by visual nutrition information and staff training to encourage healthy food consumption. As a result, low-income consumers’ food knowledge has improved and their purchases of healthy foods have increased [4]. Recently, with the establishment of pediatric-specific big data platforms, data-based research for improving children’s health is actively being conducted. For example, large-scale networks such as Genomic Information Commons (GIC), PEDSnet, PhysioNet, and PCORnet enable multi-institutional clinical research and data integration, providing important insights into the management of chronic pediatric diseases such as obesity, asthma, and rare genetic diseases. These networks have contributed to improving the diagnostic accuracy, treatment efficacy, and quality of medical services for children [5].

In the Republic of Korea, the Ministry of Food and Drug Safety (MFDS) has been operating Healthy Food Corners in convenience stores since 2022. These sections separately display and sell certified healthy products, such as low-sodium foods, fruits, vegetables, and nuts. As a result, it was found that convenience stores with Healthy Food Corners sold approximately 30% more low-sugar beverages than other stores, thereby helping to reduce sugar intake and promoting healthier eating habits among children and adolescents, who are the main users [6]. In parallel, the MFDS has been establishing the “Comprehensive Plans for Safety Management of Children’s Dietary Life” every three years since 2010 to improve children’s health and eating habits, and has been promoting various policies to create a healthy eating environment, promote the formation of eating habits, and improve food safety and nutritional levels by taking into account the changing eating environment.

These policies are closely linked to changing environmental and technological trends [7]. To reflect this effectively, data-driven trend analysis is becoming an essential tool for policy design and implementation, and extracting keywords is particularly important during trend analysis [8]. Topic modeling, one of the text-mining techniques, is a probabilistic model for extracting potential topics from large unstructured document sets, with the Latent Dirichlet Allocation (LDA) technique being a popular choice [9]. LDA is considered a valuable instrument for comprehending the policy environment and generating insights into contemporary events by examining unstructured text data (e.g., news articles) that reflect social phenomena, extracting latent themes, and providing an objective perspective on social issues [10,11]. Compared with alternative methodologies, LDA possesses the advantage of facilitating clear topic identification and intuitive interpretation. It has extensively been employed in extant research to analyze social issues and derive policy implications [12,13]. For instance, LDA-based news analysis has been employed in diverse research cases, including the identification of salient issues and healthcare industry trends [14], analysis of digital-related social issues [10], and comprehension of social perceptions concerning specific issues [15,16]. Additionally, LDA-based text mining has also been successfully applied to food safety policy analysis, providing detailed topic identification across central and local government regulations over time [17]. As such, news data analysis using text-mining techniques has widely been used to systematically identify social issues and perceptions as well as analyze potential themes embedded in articles. However, despite the increasing importance of children’s food safety, few studies have systematically analyzed how related social issues and policy discourses have evolved over time in Korea. In particular, there is a lack of research that links national dietary safety plans with real-world public discourse, such as that found in news media.

Therefore, this study aimed to comprehensively analyze the evolution of social issues related to children’s food safety in Korea by using news big data and topic modeling techniques. It focused on the five phases of the “Comprehensive Plans for Safety Management of Children’s Dietary Life” initiative, implemented from 2010 to 2024. The LDA technique was applied to derive major topics and analyze trends in each topic. Ultimately, this study sought to establish a foundation for the development of a more adaptable and efficient children’s food safety management system in a rapidly evolving technological and social landscape. Additionally, it endeavored to offer practical insights into the formulation and implementation of policies.

## 2. Materials and Methods

### 2.1. Data Collection

This study analyzed news big data to identify social issues that have primarily been discussed in Korea in relation to children’s dietary safety. News data are a resource that reflects changes in policy and social discourse over a specific period of time and can help analyze policy trends from various perspectives [18,19,20]. As illustrated in Table 1, numerous scholarly publications have employed news big data analysis to identify social issues. This study was reviewed and approved by the Institutional Review Board of Yong In University (Approval Number: 2-1040966-AB-N-01-2407-HR-352-2).

On 21 May 2024, 104 media outlets registered in BigKinds “https://www.bigkinds.or.kr (accessed on 21 May 2024)”, a news big data service of the Korea Press Foundation, were selected for analysis. BigKinds is a public big data platform that can be accessed freely by anyone upon user registration. First, 112 keywords were derived from the comprehensive plan for children’s food safety management published by the Ministry of Food and Drug Safety (1st to 5th) based on reviews by three food, nutrition, and safety experts and subsequently adopted as designated keywords. The collection period spanned from 1 January 2010, when the “Comprehensive Plans for Safety Management of Children’s Dietary Life” program was implemented, to 21 May 2024, the current research time. During this period, the designated keywords were searched. Initially, 7,140,032 news articles were collected. However, after removing 1,498,424 articles with the same publication date, media organization, and text, 5,641,608 articles were finally selected for analysis.

### 2.2. Data Analysis

#### 2.2.1. Preprocessing

A TextRank algorithm was employed to extract keywords from the articles that had been collected. The TextRank algorithm is a method of extracting keywords by calculating the frequency of the top 50 words that appear simultaneously in an article in order of weight [26]. From the final 5,641,608 articles, words that were not related to children’s food safety, such as “English,” “hello,” and “main,” were removed. Additionally, words that did not add meaning or contribute to the interpretation of the topic, such as “and,” “the,” and “this,” were removed. The final number of words selected for LDA analysis was 97,131. To refine the filtering process and enhance the quality of topic modeling, a customized stopword list, developed in addition to standard Korean NLP library stopwords (e.g., KoNLPy), was constructed and then refined through an iterative process. This process involved incorporating domain-specific function words, identifying and adding high-frequency but semantically null terms, and removing non-informative grammatical particles. The effectiveness of these customization rules was further enhanced by iteratively refining the list based on topic modeling outputs and expert feedback.

#### 2.2.2. Topic Modeling and Determination of the Number of Topics

**Figure 1:**
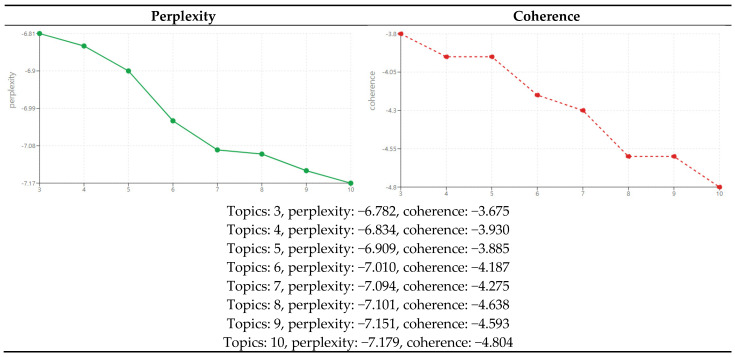
Topic perplexity and coherence.

Since the analysis target of this study was news big data, which is unstructured text data, a topic modeling technique useful for deriving latent topics was utilized. Specifically, the most broadly used LDA model was applied and analyzed using Python’s GENSIM library (version 4.3.0). The identified topics could be used to verify the proportion of keywords contributing to each topic. Additionally, to further ensure consistency and reduce subjectivity in topic labeling, the sub-task names defined in the “Comprehensive Plans for Safety Management of Children’s Dietary Life” were adopted as a theoretical framework. Each topic was named based on how its top keywords corresponded to these policy categories, as determined through expert review. This approach grounded topic labeling in institutional policy structure rather than arbitrary interpretation, thereby enhancing both theoretical rigor and practical relevance. To perform LDA analysis, the number of topics was arbitrarily determined using perplexity and coherence. However, perplexity and coherence are not absolute criteria for determining the number of topics [27]. Moreover, perceiving topic interpretation as natural storytelling by a layperson is relatively important [28]. Therefore, we examined the perplexity and coherence of each topic by applying hyperparameters from 3 to 10 topics (Figure 1), selected values approximating the optimal values to perform modeling, and repeatedly compared the results. In addition to evaluating perplexity and coherence scores, the interpretability of each topic was assessed through expert validation. Three domain experts reviewed the top keywords of each topic to confirm semantic coherence and alignment with the policy sub-goals stated in the Comprehensive Plans for Safety Management of Children’s Dietary Life. This qualitative validation step ensured that the topics were not only statistically meaningful but also policy-relevant. In this study, the optimal number of topics was determined to be seven. We organized and named the identified topics and ascertained the percentage of keywords contributing to each topic.

#### 2.2.3. Visualizing Topics: LDAvis

To help visualize the LDA modeling results, the “LDAvis” package was employed, providing the results in HyperText Markup Language format. The top 30 words displayed on the right-hand-side pane of the visualization tool were used to interpret and label the theme of each topic, with the width of the blue bar representing the overall frequency of each word and that of the red bar representing its frequency within the topic. In addition, the weight parameter λ (where 0 ≤ λ ≤ 1) can be used to determine the relevance of words and topics [29]. The closer the λ value is to 1, the more frequently the words appearing in each topic are selected as keywords. However, the closer it is to 0, the more likely it is that words with a vast difference between topics are selected (words that appear frequently within the topic). Determining the optimal value of λ for topic interpretation is difficult, and the value of λ should be adjusted to leverage its additive support for topic interpretation or labeling rather than determining the optimal λ [30]. In this study, the value of λ was set to 0.5 to account for both perspectives.

## 3. Results

The words extracted using the TextRank algorithm were divided into the five implementation phases of the “Comprehensive Plans for Safety Management of Children’s Dietary Life,” and frequency analysis revealed the top 20 words (Table 2). The top five words according to period were as follows: “campaign,” “consumer,” “online,” “convenience store,” and “students” for the first period (2010–2012); “online,” “convenience store,” “consumer,” “campaign,” and “big data” for the second period (2013–2015); “big data,” “online,” “consumer,” “convenience store,” and “campaign” for the third period (2016–2018); “online,” “big data,” “consumer,” “local government,” and “convenience store” for the fourth period (2019–2021); and “online,” “artificial intelligence,” “local government,” “consumer,” and “convenience store” for the fifth period (2022–2024). Overall, “online,” “consumer,” “convenience store,” and “students” appeared most frequently.

### 3.1. LDA Analysis Results for Child Food Safety

**Figure 2:**
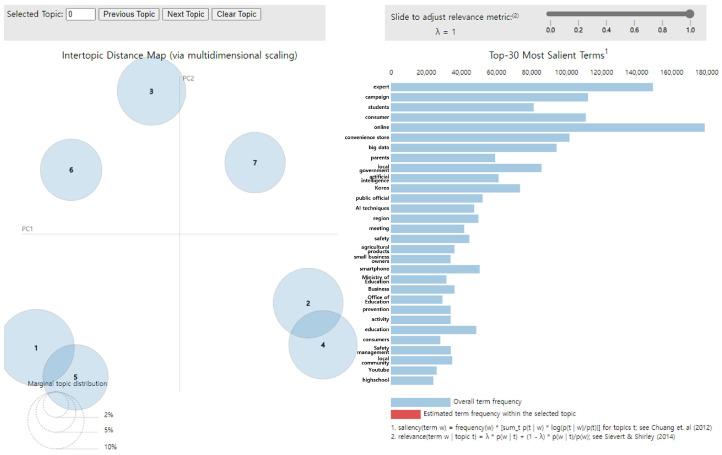
LDA analysis visualization results. Numbers 1–7 represent the topic numbers identified by the LDA model. Visualization method based on Chuang et al. [33] and Sievert & Shirley [29].

A topic modeling analysis of 97,131 keywords related to children’s dietary safety yielded seven topics, as illustrated in Figure 2. In the visualization results, the size of the circle indicates the magnitude of the topic. When a topic comprised high-frequency words, it was more likely to become the main topic. The further the distance between topics, the higher the discriminant validity and the more clearly the topics are distinguished. In contrast, if the topics were close or overlapping, discriminant validity was low; thus, they were interpreted as possessing similarity and exhibiting correlation with each other [31,32]. Specifically, topics 1 and 5 as well as topics 2 and 4 displayed considerable similarity to each other. Additionally, the topic titles were not subjectively named but rather assigned using a policy-grounded framework. Specifically, each topic was labeled based on how its constituent keywords aligned with the sub-task categories defined in the “Comprehensive Plans for Safety Management of Children’s Dietary Life,” as validated through expert review. This ensured consistency with the theoretical framework established during the modeling process.

**Figure 3:**
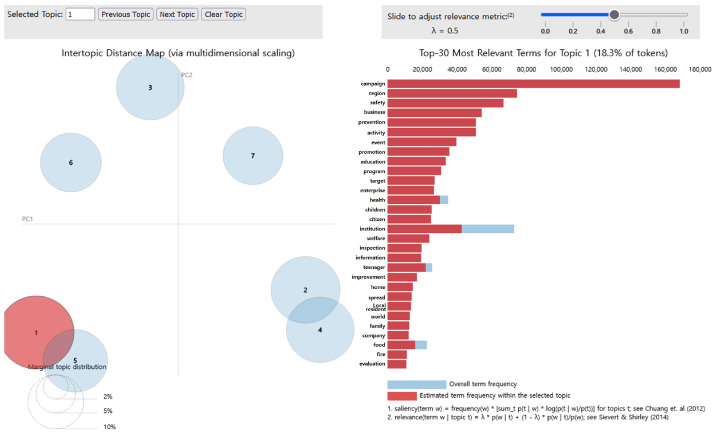
LDA analysis visualization results (Topic 1). Numbers 1–7 represent the topic numbers identified by the LDA model. Visualization method based on Chuang et al. [33] and Sievert & Shirley [29].

Among all topics, topic 1 accounted for the largest proportion (18.3%). As shown in Figure 3, the main keywords were “campaign,” “region,” “safety,” “business,” “prevention,” “activity,” “event,” “promotion,” “education,” and “program.” These top 10 words exhibited completely red bars, with no blue; therefore, they were exclusively related to topic 1. This was an excellent representation of topic 1 [30], which was subsequently named “Spread of diverse experiential education and nutrition information content.”

**Figure 4:**
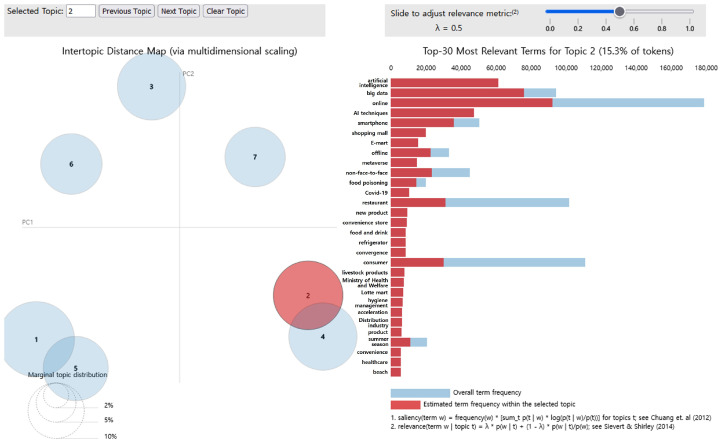
LDA analysis visualization results (Topic 2). Numbers 1–7 represent the topic numbers identified by the LDA model. Visualization method based on Chuang et al. [33] and Sievert & Shirley [29].

The main keywords for topic 2 were “artificial intelligence,” “big data,” “online,” “AI techniques,” “smartphone,” “shopping mall,” “E-mart,” “offline,” “metaverse,” and “non-face-to-face.” Topic 2 was thus named “Promotion of a data-based children’s eating habit policy” and accounted for 15.3% of all topics (Figure 4).

**Figure 5:**
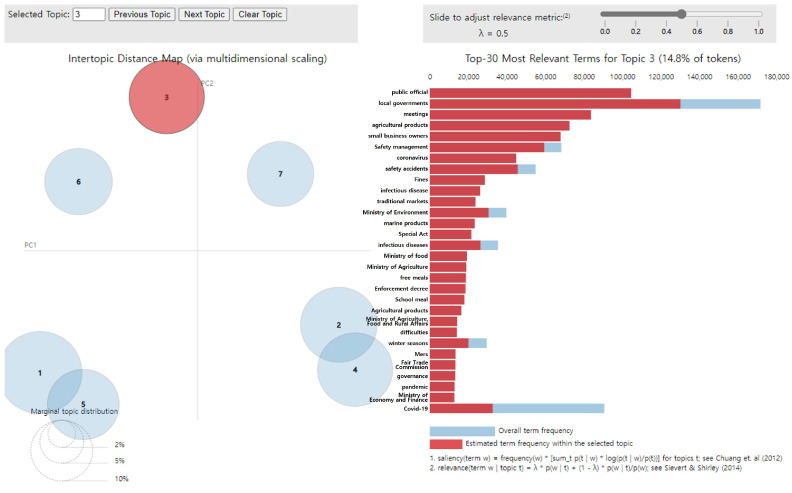
LDA analysis visualization results (Topic 3). Numbers 1–7 represent the topic numbers identified by the LDA model. Visualization method based on Chuang et al. [33] and Sievert & Shirley [29].

The main keywords for topic 3 were “public official,” “local governments,” “meetings,” “agricultural products,” “small business owners,” “safety management,” “coronavirus,” “safety accidents,” “fines,” and “infectious disease.” Hence, topic 3 was named “Management of the safety of school meals” and accounted for 14.8% of all topics (Figure 5).

**Figure 6:**
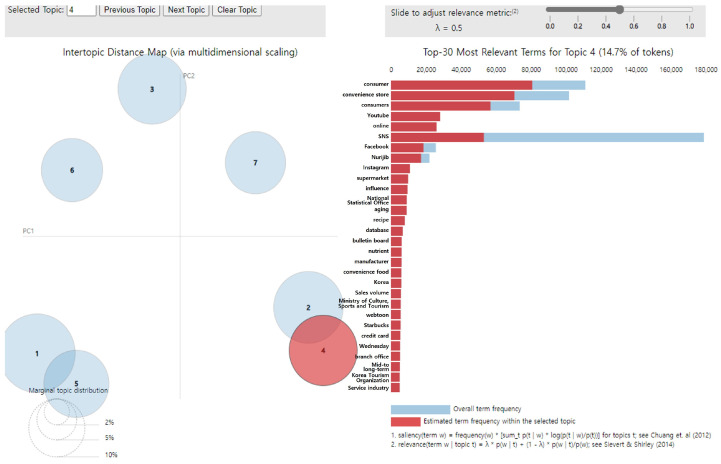
LDA analysis visualization results (Topic 4). Numbers 1–7 represent the topic numbers identified by the LDA model. Visualization method based on Chuang et al. [33] and Sievert & Shirley [29].

Topic 4 had the following main keywords: “consumer,” “convenience store,” “consumers,” “YouTube,” “online,” “SNS,” “Facebook,” “Nurijib,” “Instagram,” and “supermarket,” and it was named “Creating a healthy eating environment.” Moreover, it accounted for 14.7% of all topics (Figure 6).

**Figure 7:**
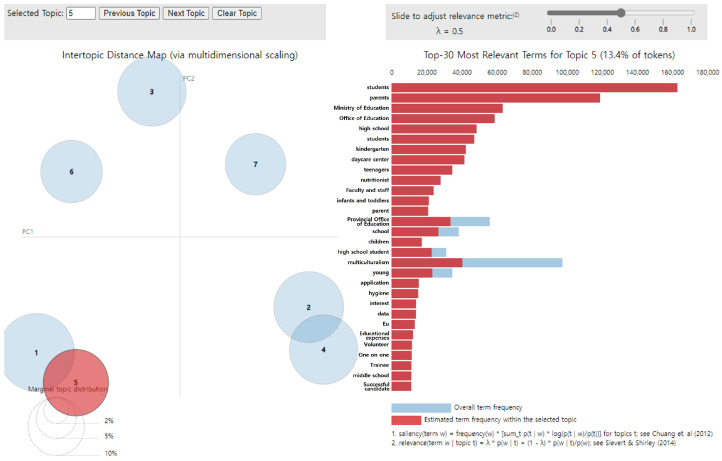
LDA analysis visualization results (Topic 5). Numbers 1–7 represent the topic numbers identified by the LDA model. Visualization method based on Chuang et al. [33] and Sievert & Shirley [29].

Under topic 5, the main keywords were “students,” “parents,” “Ministry of Education,” “Office of Education,” “high school,” “students,” “kindergarten,” “daycare center,” “teenagers,” and “nutritionist” (Figure 7). These top 10 words were exclusively associated with topic 5, as evidenced by the complete absence of blue and entire coverage of red across the bars, suggesting that the topic was effectively represented. The topic was labeled “Support for the provision of nutritional meals according to growth stage,” and it accounted for 13.4% of all topics.

**Figure 8:**
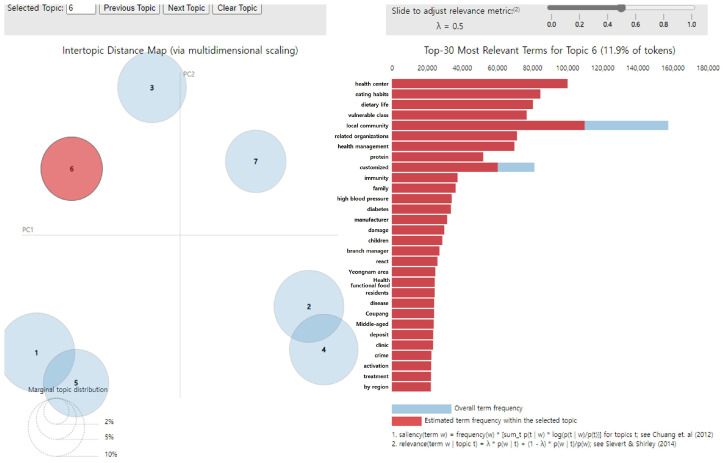
LDA analysis visualization results (Topic 6). Numbers 1–7 represent the topic numbers identified by the LDA model. Visualization method based on Chuang et al. [33] and Sievert & Shirley [29].

The main keywords for topic 6 were “health center,” “eating habits,” “dietary life,” “vulnerable class,” “local community,” “related organizations,” “health management,” “protein,” “customized,” and “immunity.” Topic 6 was thus termed “Establishing a customized safety and nutrition education system” and accounted for 11.9% of all topics (Figure 8).

**Figure 9:**
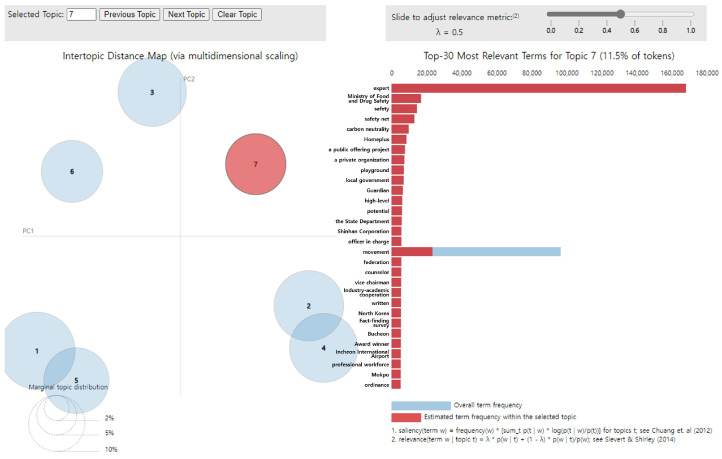
LDA analysis visualization results (Topic 7). Numbers 1–7 represent the topic numbers identified by the LDA model. Visualization method based on Chuang et al. [33] and Sievert & Shirley [29].

Topic 7 had the following main keywords: “expert,” “Ministry of Food and Drug Safety,” “safety,” “safety net,” “carbon neutrality,” “Homeplus,” “a public offering project,” “a private organization,” “playground,” and “local government” (Figure 9). These top 10 words exhibited completely red bars, with no blue; hence, they exclusively appeared in topic 7. This was an excellent representation of topic 7, which was subsequently named “Creating a private-centered food environment.” Furthermore, topic 7 accounted for 11.5% of all topics.

### 3.2. Topic-Based Analysis of Trends in the “Comprehensive Plans for Safety Management of Children’s Dietary Life”

**Figure 10:**
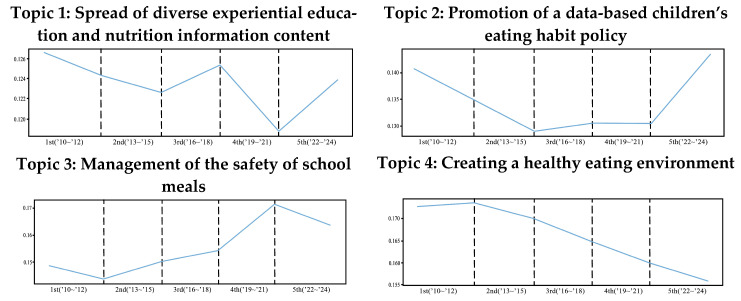

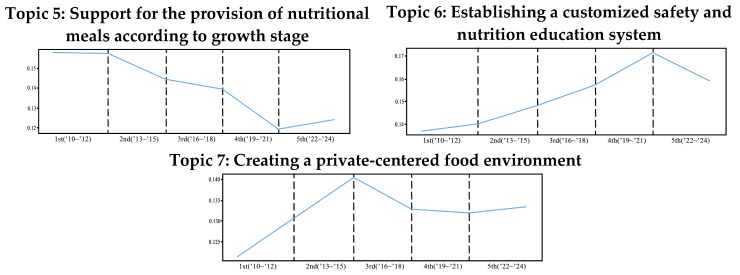
Trends in the seven topics related to children’s dietary life safety.

To elucidate shifts in the prominence of the seven topics within the “Comprehensive Plans for Safety Management of Children’s Dietary Life” initiative, the trends of major topics over time were analyzed. The trend lines for the seven topics related to children’s dietary life safety are shown in Figure 10.

“Creating a healthy eating environment (Topic 4)” displayed a continuous decrease. “Support for the provision of nutritional meals according to growth stage (Topic 5)” exhibited a gradual decrease, followed by an uptick in the fifth year. In the fifth year, “Promotion of a data-based children’s eating habit policy (Topic 2)” demonstrated a sharp increase, whereas “Establishing a customized safety and nutrition education system (Topic 6)” displayed a decreasing trend. “Creating a private-centered food environment (Topic 7)” slightly decreased in the third year but exhibited a steady increase thereafter.

## 4. Discussion

To identify policy trends related to children’s dietary safety, this study collected news articles from 2010 to 2024 during the “Comprehensive Plans for Safety Management of Children’s Dietary Life” period and performed LDA analysis during text mining. This facilitated the identification of transitions in policy and social interest over time as well as the discernment of key trends.

First, an analysis of words appearing in each of the five implementation phases of the “Comprehensive Plans for Safety Management of Children’s Dietary Life” program revealed that data-based keywords, such as “big data,” “artificial intelligence,” and “AI techniques,” remained in the top 10 from the third phase and tended to continuously rise in ranking. This finding suggests an increasing interest in data-based customized policy support [34,35]. In the Republic of Korea, prominent government agencies, such as the Ministry of Food and Drug Safety; Ministry of Agriculture, Food, and Rural Affairs; Ministry of Education; Ministry of Oceans and Fisheries; Ministry of the Interior and Safety; and Rural Development Administration, are constructing a standardized food and nutrition information and integrated database with the goal of improving the eating habits of children and adolescents and preventing obesity and nutritional imbalances. In addition, statistical data from each ministry (e.g., data generated from the Student Health Behavior Survey, Food Consumption Behavior Survey, Dietary Lifestyle Safety Index, and National Health and Nutrition Survey) are being utilized to improve policy efficiency [36]. In addition, the Ministry of Health and Welfare developed an information and communication technology-based public mobile healthcare service model in 2016 to provide non-face-to-face health consulting for adults facing health risks; since 2022, the ministry has been expanding this service to provide data-driven, personalized health guidance to elementary and middle school students [37]. This trend is also consistent with international policy trends. For example, the European Union is actively using digital food environment analysis in policy decision-making and is promoting interventions to improve children’s dietary lifestyles through data-based policies [3]. In addition, an international conference (INNAN 2025) emphasized the need for global cooperation to strengthen data-based nutrition policies, which are requisite to improving multinational nutrition policies and public health policies [38]. Furthermore, as the importance of data-based policies has become increasingly evident following the COVID-19 pandemic, data-based strategies that address food insecurity and nutritional imbalances are being investigated from multiple angles and reflected in actual policies [39].

“Convenience store” consistently appeared across all five implementation phases, suggesting that convenience stores have become the predominant source of snacks and meals for children [40,41,42]. In response to these changes, the Republic of Korea introduced the “Healthy Food Corner” project in 2022, centered around convenience stores near schools, facilitating children’s access to reduced-sodium kimbap and low-sugar drinks, among others [6]. Since 2014, the United States has been promoting healthy food consumption in small stores through the HCSN project [4], while the United Kingdom has significantly increased the sales of healthy foods and consumption of fresh foods in convenience stores in poor areas through the Change4Life program [43].

Meanwhile, “Campaign” exhibited a consistent top-tier ranking throughout the five implementation phases, despite demonstrating a gradual decline. In contrast, “Online” received consistent emphasis, securing a top-three position across all phases. This indicates that the promotion of dietary life safety management policies online as well as offline has become an important means of policy implementation, suggesting the need for the digital transformation of policies. A previous study substantiated the necessity for digital transformation by examining the efficacy of online marketing regulations for food and non-alcoholic beverages intended for children [44]. Consequently, it would be prudent for future dietary life safety management policies to evolve in a direction that integrates both online and offline aspects.

On analyzing the trend of each topic within the “Comprehensive Plans for Safety Management of Children’s Dietary Life” program, “Creating a healthy eating environment (Topic 4)” continuously decreased with time. This is a result that is in contrast to the research results of Lee HS & Kim JH (2021) [45], such as the increase in online purchases and the expansion of convenience store use, and policy supplementation that reflects the change in the consumption environment is required [46]. Conversely, “Support for the provision of nutritional meals according to growth stage (Topic 5)” gradually decreased, followed by an uptick in the fifth year. This phenomenon presumably emanated from a reexamination of nutrition, safety, and meal-related issues as school meals resumed following the pandemic. In addition, whereas “Promotion of a data-based children’s eating habit policy (Topic 2)” increased rapidly in the fifth year, “Establishing a customized safety and nutrition education system (Topic 6)” exhibited a decreasing trend. This suggests that customized support can be realized more effectively when numerous data are generated. Therefore, in future policies, strengthening the data-based system is requisite to promoting more sophisticated, customized support. In particular, establishing a more precise regulatory response strategy by utilizing real-time consumption data analysis is imperative. In Europe, analytics of the digital food environment have been used to inform policy interventions that promote healthy eating habits in children, most notably, regulation of the online advertising of ultra-processed foods and strengthening of healthy food labeling policies [3,44]. Meanwhile, “Creating a private-centered food environment (Topic 7)” slightly decreased in the third phase but displayed a steady increase thereafter. Therefore, to improve children’s dietary lifestyles, research on the surrounding and consumption environments is necessary in addition to school meals; moreover, collaboration and programming with private organizations are important, not merely at the school level.

However, this study has certain limitations. First, the analyzed data were constrained to domestic news articles, thus not fully reflecting linkages with international dietary policies and trends. Since children’s dietary lifestyles are influenced by international exchanges and trends [47,48], future studies require expanded analyses that include overseas news and policies. Second, this study exclusively analyzed articles registered in BigKinds, a news big data service of the Korea Press Foundation. Consequently, media bias and editorial policies might have affected the analysis results. Furthermore, the number of articles might have increased or decreased depending on social issues at certain times. In subsequent studies, mitigating data bias and enhancing study reliability by concurrently analyzing news data from multiple sources or incorporating additional data, such as policy documents and social opinion survey data, into the analysis are imperative. Third, while this study effectively identified major policy topics through LDA analysis, further interpretation is required to explain how these topics reflect broader societal and economic shifts. For example, the analysis period (2010–2024) includes the COVID-19 pandemic, which significantly impacted global and national policy priorities, especially regarding public health and fiscal resource allocation. During this time, many governments, including Korea, experienced reduced fiscal space, which may have constrained funding for non-COVID-related projects, including child food safety initiatives [49,50]. In addition to these public funding constraints, when fiscal deficits are elevated the credit risk faced by banks increases, which in turn leads to a decline in loan supply; this dynamic can further restrict capital availability for food safety- and sustainability-related projects [51]. Simultaneously, the private sector also restructured investment portfolios, favoring more flexible, innovation-oriented strategies over capital-intensive, irreversible projects [52,53,54]. This shift could have influenced the types of food safety projects pursued by non-governmental actors. Moreover, the global rise in ESG (Environmental, Social, and Governance) investing during this period likely redirected private capital toward socially responsible sectors, such as children’s health and nutrition [55]. This may explain the increasing frequency of sustainability- and equity-related keywords observed in recent phases of the topic analysis. Accordingly, the findings of this study should be interpreted not only as a reflection of policy content but also as an indicator of how public discourse and institutional priorities respond to geopolitical shocks, budgetary constraints, and emerging investment trends.

Nonetheless, the present study is noteworthy for its systematic, data-based analysis of major trends related to children’s dietary life safety management according to period. Notably, the study holds both academic and practical value as it derived policy implications by utilizing news big data and LDA techniques, yielding basic data for policy establishment and implementation in a changing environment. Given the limitations identified, future research should incorporate a broader set of data sources, including policy documents, stakeholder interviews, and social opinion datasets, to ensure a more comprehensive understanding. In terms of policy application, it is also essential to complement statistical analyses with expert validation to enhance contextual relevance. Policymakers are thus encouraged to design adaptive strategies that align with evolving public and private sector dynamics—such as real-time data monitoring, fiscal reallocation, and ESG-driven investments—to build a resilient and responsive children’s dietary life safety system. Future research can thus further develop a children’s dietary life safety management system by specifically interpreting trends and international linkages.

## 5. Conclusions

This study provides valuable insights for researchers, practitioners, and policymakers in the field of children’s food safety management. The findings underscore the importance of data-driven policy development in response to technological advancements and evolving food consumption behaviors. The increasing application of AI and big data analytics in policy discussions presents novel opportunities for integrating technology into dietary safety regulations more effectively.

By leveraging data-driven insights, policymakers and practitioners can develop adaptive interventions that reflect real-time consumption patterns. International studies have demonstrated that collaboration among government agencies, the private sector, and educational institutions plays a crucial role in enhancing children’s dietary education and strengthening food safety frameworks.

To explore the impact of digital environments, especially online food retailing, on children’s dietary habits and food safety, further research is warranted. A more comprehensive understanding of these trends will help formulate evidence-based strategies that foster safer and healthier food environments for children.

## Figures and Tables

**Table 1:** Articles analyzing news big data using topic modeling.

Author	Year	Article
Ittefaq M, Zain A, Arif R, Ala-Uddin M, Ahmad T, Iqbal A [21]	2025	Global news media coverage of artificial intelligence (AI): A comparative analysis of frames, sentiments, and trends across 12 countries
Chen S, Ngai CSB, Cheng C, Hu Y [22]	2025	Analyzing Themes, Sentiments, and Coping Strategies Regarding Online News Coverage of Depression in Hong Kong: Mixed Methods Study
Choi YJ, Um YJ [23]	2023	Topic Models to Analyze Disaster-Related Newspaper Articles: Focusing on COVID-19
Kim SY [10]	2023	Discovering Policy Implications from Analysis of News Big-data Related to Digital Issues Based on LDA Topic-modeling
Seo JW, Koh SK [24]	2023	Trends in the issues of housewives in newspaper articles using topic modeling based on big data
Cha YR [25]	2023	Big Data Analysis of Metaverse and Advertising related to News Articles: Focusing on Topic Modeling

**Table 2:** Top 20 words appearing in each of the five implementation phases.

No.	1st (2010–2012)		2nd (2013–2015)		3rd (2016–2018)		4th (2019–2021)		5th (2022–2024)	
	Keyword	*N*	Keyword	*N*	Keyword	*N*	Keyword	*N*	Keyword	*N*
1	campaign	28,388	online	51,204	big data	51,208	online	140,490	online	80,583
2	consumer	27,527	convenience store	36,719	online	46,808	big data	68,375	artificial intelligence	59,140
3	online	27,355	consumer	36,692	consumer	42,639	consumer	59,586	local government	52,477
4	convenience store	23,358	campaign	34,916	convenience store	41,637	local government	54,536	consumer	45,284
5	students	17,725	big data	22,962	campaign	36,705	convenience store	51,543	convenience store	42,840
6	local government	14,675	local government	22,401	local government	30,970	artificial intelligence	43,520	big data	41,783
7	support	10,457	students	21,712	students	24,216	campaign	43,166	parents	38,824
8	agricultural products	9760	support	13,200	artificial intelligence	19,452	students	37,333	students	34,568
9	parents	9464	parents	11,411	support	17,067	AI techniques	35,296	campaign	33,816
10	education	8939	safety	11,340	AI techniques	15,632	parents	26,530	AI techniques	30,172
11	medicine	8602	agricultural products	11,220	safety	13,280	support	23,211	supply chain	20,011
12	management	8329	safety management	10,866	education	13,204	infection	21,743	support	19,885
13	participation	7390	education	10,714	parents	12,563	local community	20,525	local community	19,098
14	safety	7387	Mers	10,672	safety management	12,481	infectious diseases	20,516	safety management	17,827
15	free meals	7231	management	10,355	agricultural products	11,650	agricultural products	19,870	education	16,578
16	prevention	6945	medicine	9605	management	11,511	education	17,021	safety accident	14,243
17	eating habits	5888	prevention	9371	medicine	10,937	medicine	15,901	agricultural products	14,057
18	school	5866	institutes street	9198	local community	10,765	safety management	15,729	safety	13,813
19	cooperation	5841	safety accident	8520	prevention	10,016	safety	15,053	medicine	12,998
20	children	5770	participation	8503	participation	9900	high school	13,472	high school	12,527

## Data Availability

The data presented in this study are openly available in BigKinds “https://www.bigkinds.or.kr (accessed on 21 May 2024)”, a public news database operated by the Korea Press Foundation. Access is free upon user registration.

## Abbreviations

The following abbreviations are used in this manuscript:

AI	Artificial Intelligence
LDA	Latent Dirichlet Allocation

## References

[1] Shim J.E. (2021). Picky Eating and Factors Affecting Food Acceptance. J. East Asian Soc. Diet. Life.

[2] Greenthal E., Marx K., Friedman E., John S., Johnson J., LiPuma C., Nara D., Sorscher S., Gardner K., Musicus A. (2024). Navigating the online food environment: Policy pathways for promoting food access, transparency, and healthy food choices online. Front. Nutr..

[3] Granheim S.I., Løvhaug A.L., Terragni L., Torheim L.E., Thurston M. (2022). Mapping the digital food environment: A systematic scoping review. Obes. Rev..

[4] Paluta L., Kaiser M.L., Huber-Krum S., Wheeler J. (2019). Evaluating the impact of a healthy corner store initiative on food access domains. Eval. Program Plann..

[5] Vesoulis Z.A., Husain A.N., Cole F.S. (2023). Improving child health through Big Data and data science. Pediatr. Res..

[6] Ministry of Food and Drug Safety (MFDS) (2023). Report on the Pilot Operation of Health Food Corners in Convenience Stores.

[7] Joamets K. (2024). Children’s right to healthy food and the digital market—Need for legal and policy development. Balt. J. Eur. Stud..

[8] Baranowski M. (2022). Epistemological aspect of topic modelling in the social sciences: Latent Dirichlet Allocation. Przegląd Kryt..

[9] Jang S.Y., Jung S.H. (2021). An Analysis of the Research Trends for Urban Study using Topic Modeling. J. Korea Acad.-Ind. Coop. Soc..

[10] Kim S.Y. (2023). Discovering Policy Implications from Analysis of News Big-data Related to Digital Issues Based on LDA Topic-modeling. Korean Public Adm. Q..

[11] Alhashmi M., Maree M., Saadeddin Z. (2021). Using Latent Dirichlet Allocation and Text Mining Techniques for Understanding Medical Literature. Int. J. Comput..

[12] Park S.K., Lee B.G. (2019). A Text Mining Approach to the Analysis of Issues for Accommodation Sharing Business. J. Tour. Leis. Res..

[13] Kim K.H., Jun C.S., Song C.H., Jeon J.H. (2024). Patent Trend Analysis of Unmanned Ground Vehicles(UGV) using Topic Modeling. J. KIMST.

[14] Kim E.J., Choi H.J. (2022). Analyzing Core Tehnology and Technological Convergence in Healthcare Using Topic Modeling and Network Analysis: Focus on Patent Information. J. Korea Inst. Inf. Commun. Eng..

[15] Lee S.S., Yoo I.H., Kim J.H. (2020). An analysis of public perception on Artificial Intelligence (AI) education using Big Data: Based on News articles and Twitter. J. Digit. Converg..

[16] Song C., Guo C., Hunt K., Zhuang J. (2020). An Analysis of Public Opinions Regarding Take-Away Food Safety: A 2015–2018 Case Study on Sina Weibo. Foods.

[17] Song C., Guo J., Gholizadeh F., Zhuang J. (2022). Quantitative Analysis of Food Safety Policy—Based on Text Mining Methods. Foods.

[18] Barkho L. (2023). For a postfoundational method to news discourse analysis. Cogent Arts Humanit..

[19] Park S.K., Lee H.J., Lee B.G. (2021). Exploring Social Issues of On-demand Delivery Platform Participants. J. Digit. Converg..

[20] Gebhard L., Hamborg F. (2020). The POLUSA Dataset: 0.9M Political News Articles Balanced by Time and Outlet Popularity. Proceedings of the ACM/IEEE Joint Conference on Digital Libraries in 2020.

[21] Ittefaq M., Zain A., Arif R., Ala-Uddin M., Ahmad T., Iqbal A. (2025). Global news media coverage of artificial intelligence (AI): A comparative analysis of frames, sentiments, and trends across 12 countries. Telemat. Inform..

[22] Chen S., Ngai C.S.B., Cheng C., Hu Y. (2025). Analyzing Themes, Sentiments, and Coping Strategies Regarding Online News Coverage of Depression in Hong Kong: Mixed Methods Study. J. Med. Internet Res..

[23] Choi Y.J., Um Y.J. (2023). Topic Models to Analyze Disaster-Related Newspaper Articles: Focusing on COVID-19. Int. J. Ment. Health Promot..

[24] Seo J.W., Koh S.K. (2023). Trends in the issues of housewives in newspaper articles using topic modeling based on big data. J. Fam. Better Life.

[25] Cha Y.R. (2023). Big Data Analysis of Metaverse and Advertising related to News Articles: Focusing on Topic Modeling. J. Pract. Res. Advert. Public Relat..

[26] Cho S.G., Park H.J. (2023). Analysis of Consumer Food Safety Issues Due to COVID-19.

[27] Park G.T., Im S.H., Kim M.S., Choi D.H., Song B.M. (2022). Analysis of Key Topics in Green Logistics Using LDA—Focusing on Keywords Before and After the COVID-19. J. Korean Prod. Oper. Manag. Soc..

[28] Park S.U., Kang J.Y., Jung S.C. (2022). Complete Guide to Python Text Mining: From Natural Language Processing Basics to Deep Learning-Based BERT Models.

[29] Sievert C., Shirley K. LDAvis: A Method for Visualizing and Interpreting Topics. Proceedings of the Workshop on Interactive Language Learning, Visualization, and Interfaces.

[30] Gencoglu B., Helms-Lorenz M., Maulana R., Jansen E.P., Gencoglu O. (2023). Machine and expert judgments of student perceptions of teaching behavior in secondary education: Added value of topic modeling with big data. Comput. Educ..

[31] Ferdinand B.J., Aviarta N.P., Jordan M.G., Purwandari K. Topic Identification of Science and Mathematics Literature Using Latent Dirichlet Allocation. Proceedings of the 2024 IEEE International Conference on Artificial Intelligence and Mechatronics Systems (AIMS).

[32] Kim H.O., Park H.S., Hong D.Y. (2024). Subalternity of Refugee Women: Focused on the Semantic Connectivity and Topic Analysis of English Literature on Refugee Women. J. Crit. Soc. Welf..

[33] Chuang J., Manning C.D., Heer J. Termite: Visualization techniques for assessing textual topic models. Proceedings of the International Working Conference on Advanced Visual Interfaces.

[34] de Sousa R.F., Balcerzak A., Bevere T., de Quadros V.P. (2020). FAO/WHO GIFT: Increasing the availability, harmonization and use of individual quantitative food consumption data worldwide. Eur. J. Public Health.

[35] Barnes C., Yoong S.L., Nathan N., Wolfenden L., Wedesweiler T., Kerr J., Ward D.S., Grady A. (2021). Feasibility of a Web-Based Implementation Intervention to Improve Child Dietary Intake in Early Childhood Education and Care: Pilot Randomized Controlled Trial. J. Med. Internet Res..

[36] Ministry of Food and Drug Safety (2025). The 5th Children’s Dietary Safety Management Comprehensive Plan (2022~2024).

[37] Kim H.Y., Kim D.J., Lee Y.S., Lim S.T., Cho J.H. (2023). The Effect of Participation in Public Health Center Mobile Health Care Service for on Health Physical Fitness of Elementary-Middle Students. Korean J. Growth Dev..

[38] Laur C., Johnsen J.T., Bradfield J., Eden T., Mitra S., Ray S. (2020). Closing the Gap: Data-Based Decisions in Food, Nutrition and Health Systems: Proceedings of the Fifth International Summit on Medical and Public Health Nutrition Education and Research. BMJ Nutr Prev. Health.

[39] Laperriere A., Bohn J., do Vale M.L. (2022). 5 Data-Driven Action for Food Systems Transformation. BMJ Nutr. Prev. Health.

[40] Kim S.Y. (2021). A Study of Food Behavior and Food Purchasing Behavior of High School Students According to Frequency of Use of Convenience Stores. Master’s Thesis.

[41] Park H., Kang H., Lee E.S., Lee H. (2021). The snacking pattern, diet, lifestyle and menu preferences of elementary school students in Gyeonggi area, considering the most frequently eaten snacks. J. Nutr. Health.

[42] Rhodes D., Morton S., Moshfegh A. (2021). Convenience Stores: Source of Food/Beverages Among Children, What We Eat in America, NHANES, 2015–2018. Curr. Dev. Nutr..

[43] Department of Health of UK (2010). Change4Life Convenience Stores Evaluation Report 2010.

[44] Boyland E., McGale L., Maden M., Hounsome J., Boland A., Jones A. (2022). Systematic review of the effect of policies to restrict the marketing of foods and non-alcoholic beverages to which children are exposed. Obes. Rev..

[45] Lee H.S., Kim J.H. (2021). Analysis of Food Consumption Behavior due to COVID-19: Focusing on MZ Generation. J. Digit. Converg..

[46] Shen C., Wei M., Sheng Y. (2021). A bibliometric analysis of food safety governance research from 1999 to 2019. Food Sci. Nutr..

[47] Saavedra J.M. (2022). The Changing Landscape of Children’s Diet and Nutrition: New Threats, New Opportunities. Ann. Nutr. Metab..

[48] Salmon K., Shin D., Kmush B., Wallia B., Bellows A., Larsen D. (2020). Evidence of declining dietary diversity among children aged 1–5 years between 2005 and 2017 in lower-income countries. Curr. Dev. Nutr..

[49] Eichenbaum M., Rebelo S., Trabandt M. *Epidemics in the Neoclassical and New-Keynesian Models*; NBER Working Paper; National Bureau of Economic Research: Cambridge, MA, USA, 2020; p. 2.

[50] Campello M., Kankanhalli G., Muthukrishnan P. (2023). Corporate hiring under COVID-19: Financial constraints and the nature of new jobs. J. Financ. Quant. Anal..

[51] Silva F.B.G. (2021). Fiscal Deficits, Bank Credit Risk, and Loan-Loss Provisions. J. Financ. Quant. Anal..

[52] Bloom N. (2009). The impact of uncertainty shocks. Econometrica.

[53] Campello M., Cortes G.S., d’Almeida F., Kankanhalli G. (2022). Exporting uncertainty: The impact of Brexit on corporate America. J. Financ. Quant. Anal..

[54] Campello M., Kankanhalli G., Kim H. (2024). Delayed creative destruction: How uncertainty shapes corporate assets. J. Financ. Econ..

[55] Dantas M. (2021). Are ESG Funds More Transparent?. SSRN.

